# Caudal block with rectal diclofenac and paracetamol for pediatrics infra umbilical surgery at a comprehensive specialized teaching hospital in Ethiopia

**DOI:** 10.1016/j.amsu.2020.11.071

**Published:** 2020-11-28

**Authors:** Dereje Zewdu, Misrak WoldeYohannis, Fissiha Fentie, Abdisa Aga, Assefa Hika, Diriba Teshome

**Affiliations:** a Department of Anesthesia, College of Health Science and Medicine, Diredawa University, Diredawa, Ethiopia; b Department of Anesthesia, College of Health Science and Medicine, Addis Ababa University, Addis Ababa, Ethiopia; c Department of Anesthesia, Harar College of Health Science, Harar, Ethiopia; d Department of Anesthesia, College of Health Science and Medicine, Aksum Tsion University, Aksum Tsion, Ethiopia; e Department of Anesthesia, College of Health Science and Medicine, Debre Tabor University, Debre Tabor, Ethiopia

**Keywords:** Caudal block, Diclofenac, Paracetamol, Postoperative analgesia

## Abstract

**Background:**

Caudal block is a common regional technique performed for infra umbilical surgery in pediatrics. Its limited duration of analgesia remains a gap in routine clinical practice. This study aimed to assess the analgesic effectiveness of caudal block with rectal diclofenac or rectal paracetamol among pediatric patients who underwent infra umbilical surgery.

**Methods:**

A prospective cohort study was conducted on patients aged 1–10 years that underwent elective infra umbilical surgery. Patients were allocated into the Caudal block with rectal Diclofenac, Caudal block with rectal Paracetamol, and Caudal block alone groups based on a postoperative pain management plan. Analysis of variance was used for normally distributed data and the Kruskal Wallis H test was used for non-normally distributed. The Tukey for post hoc test was used to compare the difference between groups one with the others. Categorical data were analyzed by using Pearson Chi-squared or Fisher's exact test as appropriate. A p-value < 0.05 considered as statistically significant.

**Results:**

The postoperative median pain score was lower in CD compared to CP and CA group (p-value < 0.001) at the 4th and 8th hour. Time to first analgesic request was significantly longer within CD 735 (540–1200 min) compared to CP 445 (240–840 min p = 0.029) and CA 315 (240–720 min p < 0.001).

**Conclusion:**

The pain score and total postoperative analgesic consumption were significantly reduced in addition to prolonged-time to request the first analgesia in the CD group compared to CA and CP group.

## Highlights

• Caudal block with rectal diclofenac might reduce analgesic request.
• Caudal block with rectal diclofenac might prolong analgesia duration.
• Caudal block with rectal diclofenac might reduce pain severity score.

## 1 Introduction

Postoperative pain in pediatric patients who underwent surgery is usually underestimated and undertreated [1]. However, a declaration of Montreal states “Access to Pain Management is a Fundamental Right”. While 80% of people worldwide do not receive adequate treatment for pain [2]. Based on 2019, a prospective longitudinal study done in Ethiopia the prevalence of moderate to severe postoperative pain was 88.2%, and of those 58.4% were inadequately treated [3]. The provision of adequate postoperative pain management not only minimizes patient suffering but also reduces morbidity, facilitates rapid recovery and early discharge from the hospital [4].

Alternatives to improve analgesia effectiveness of this block is to use caudal catheter may help to provide continuous analgesia for infra umbilical procedures in children, but affect postoperative mobility and carry the risk of infection [5,6]. In another way, Opioids are effective analgesia for postoperative pain but they are commonly associated with respiratory depression, itching, nausea, and vomiting [7].

There are different studies done in different clinical setup and countries which compare the analgesic effectiveness of caudal block combined with rectal diclofenac (CD), caudal block combined with rectal paracetamol (CP) and caudal block alone (CA) as a part of postoperative analgesia for infra-umbilical surgery in pediatrics. But there were conflicting results regarding the intensity to reduce pain severity [[8], [9], [10], [11], [12], [13], [14], [15]].

Hence, the primary outcome of this study is to compare the pain severity score of Face, Legs, Activity, Cry, and Consolability/Numeric Rating scale (FLACC/NRS) between CA, CP, and CD for infra umbilical procedure under general anesthesia. The secondary outcomes are to compare first analgesia request time and total analgesic consumption within 24 h of the postoperative period between CA, CP, and CD.

## 2 Materials and methods

### 2.1 Study design, area, and patients

A Hospital-based prospective cohort study was conducted at Tikur Anbessa Specialized Hospital from January 2019 to April 2019. XX hospital is one of the leading teaching Hospitals in Addis Ababa, the capital city of Ethiopia. Informed consent was taken from a parent of the study participants after telling them the aim of the study, benefit, harm of participating in the study, and they have been told as they can withdraw from the study at any step if they feel so. Confidentiality was secured at every step of the study. This study is reported in line with STROCSS criteria [16] and registered at www.researchregistry.com with Research Registry UIN: researchregistry6238, and available: https://www.researchregistry.com/browse-the registry#home/registrationdetails/5fa9102a5030b800153ebb76/

### 2.2 Sample size and sampling procedures

The outcome measure of our study was to compare pain severity by FLACC/NRS score, time to first analgesic request, total analgesic consumption, and incidence of adverse effects between groups within 24 h postoperative period. Sample size estimation was determined by using a priori power analysis (G Power version 3.1.9.2) based on the results of a similar study performed by Nnaji et al. [10] in Nigeria; first analgesia request time (12.93 ± 4.46 h) in CD, (7.75 ± 3.12 h) in CP and (6.43 ± 2.94 h) in CA groups and pooled standard deviation would be 3.42. Controlling for the probability of a Type I error at alpha = 0.017 (the alpha level was reduced using a Bonferroni correction, 0.05/3 = 0.017, to allow for comparisons of both exposed group with the non-exposed group), a sample of 31 subjects per group would have 80% power to detect a difference between groups. The calculated sample size was 84; by adding a 10% attrition rate and assuming a balanced design the total sample size was 93. A situational analysis was done depend on average values of previous surgery per 3 months on the logbook, 189 patients were operated on pediatric elective infra-umbilical surgery under the caudal block. A systematic random sampling technique was used to select study participants. The sampling interval k was determined by using the formula: k = N/n; where, n = total sample size, N = population per 3 months. Accordingly, 93 participants were recruited with a probability of about 49.2%. Therefore, the sampling interval is 2 and the first study participant (random start) was selected using the lottery method after which the data collector recruited 1 patient for every 2 consecutive patients undergone Infra umbilical surgery. Depend on their exposure status patients were assigned to three groups.

#### 2.2.1 Inclusion criteria

Pediatric elective patients, ASA physical status I and II, 1–10 years old age, who were received caudal block, and underwent infra-umbilical surgeries were included in the study.

#### 2.2.2 Exclusion criteria

Failed caudal block, day case surgery, additives added, caudal bupivacaine other than 0.25% concentration, and 1 ml/kg dose were excluded from the study.

### 2.3 Data collection

After providing training for data collectors, data was collected using pretested questionnaires with multiple close-ended questions. Children to take part in the study were assessed before surgery following verbal and written informed consent was taken from the family. On the morning of the surgery a trained data collector instructed the patient whose age was >5 on how to self-report pain using the eleven-point NRS score (0–10) and <5 years was assessed by FLACC score. Baseline vital signs, Induction, incision, and CB time were documented. Pre-incision vital signs were measured 10 min after the block just before skin incision. Post incision vital signs were measured 10 min after skin incision, then, the Ability to Maintained value as compared to values before incision indicates a successful block. Intra-operative data was collected by anesthetists while postoperative data was collected by four trained nurses and the PI was supervised the completeness of the data daily. Vital signs were recorded on admission to PACU and then every 20 min till the patient was discharged to the ward. FLACC/NRS scale was used to assess postoperative pain, based on the age of patients. A score of greater than 3 indicated pain. Those children were given rescue analgesia. Patients were observed by trained nurses & pain score was documented at PACU, 2nd, 4th, 8th, 12th, and 24th postoperative hours. Analgesic consumption, analgesia duration, and adverse effects were documented when it was reported within 24 h during the post-operative period.

### 2.4 Anesthesia care

In the study area, the routine practice of intraoperative and postoperative pain management for the infra-umbilical procedure in pediatrics are provided by 0.25% of 1 ml/kg caudal bupivacaine alone or caudal bupivacaine combined with 1 mg/kg rectal diclofenac and 30 mg/kg rectal paracetamol depend on preference and decision of responsible anesthetist.

On the arrival of patients to the operation theater, standard monitoring protocol including a pre-cordial stethoscope, noninvasive blood pressure, and pulse oximetry have been recorded. General anesthesia was induced by either Propofol 2–3 mg/kg or ketamine 1–2 mg/kg and with or without Suxamethonium 1–2 mg/kg.to facilitate tracheal intubation and Laryngeal mask airway insertion respectively. Maintenance anesthesia was used by either Halothane or Isoflorane.

After securing the airway, patients were assigned to Group I CA (0.25% of 1 ml/kg caudal bupivacaine alone), Group II CD (0.25% of 1 ml/kg caudal bupivacaine combined with rectal diclofenac1mg/kg), and Group III CP (0.25% of 1 ml/kg caudal bupivacaine combined with rectal paracetamol 30 mg/kg) were administered based on the bodyweight of child 10–20 min before the start of surgical incision depending on the decision of anesthetist in charge.

### 2.5 Data analysis

Data was analyzed using statistical package for Social Sciences (SPPS) software Version 20. The data were tested for normality using the Shapiro Wilk test. Levene's test was used to check Homogeneity of variance. Numeric data were expressed as a mean and standard deviation (SD) for normally distributed and median (Interquartile range) for non-normally distributed. Analysis of variance (ANOVA) was used for normally distributed data and the Kruskal Wallis H test was used for non-normally distributed or non-parametric data. If these ANOVA and Kruskal Wallis H tests were significant, the Tukey post hoc test was used to compare the difference between groups one with the others. Categorical data were analyzed by using Pearson Chi-squared or Fisher's exact test as appropriate. A p-value < 0.05 considered as statistically significant.

### 2.8 Operational definitions

FLACC scale: is a measurement used to assess pain for children between ages two months and five years or individuals that are unable to communicate their pain going to score [17].

NRS: is a valid pain intensity assessment tool that involves asking a patient to rate his or her pain from 0 to 10 (11-point scale) with the understanding that 0 NRS is equal to no pain and 10 NRS equal to the worst possible pain [18].

Time to first analgesia request: a time in minutes from the caudal placement of drug till the first recording of FLACC/NRS score >3.

## 3 Results

### 3.1 Demographic and peri-operative characteristics

Ninety (90) patients (30 each group) completed the follow-up and analyzed. Demographic and perioperative characteristics are comparable between the groups p-value >0.05 (Table 1).

**Table 1:** Demographic and perioperative characteristics of the study participants.

Variables	Group CA	Group CP	Group CD	P-value
Age (year)^a^	4.96 ± 2.3	4.63 ± 2.25	4.46 ± 2.26	0.97
Gender (M/F)^b^	21/9	19/11	23/7	0.530
ASA I/II^b^	26/4	27/3	25/5	0.749
Weight (kg)^a^	13.96 ± 2.63	13.46 ± 2.69	14.1 ± 2.34	0.769
Baseline Heart Rate	129 ± 10.49	131 ± 11.38	133.5 ± 11.93	0.827
Baseline MAP	60.17 ± 5.37	63.96 ± 3.4	62.97 ± 4.13	0.231
Atropine pre medication	16/14	18/12	20/10	0.574
Induction agent^b^ Ketamine/Propofol	12/26	15/15	14/16	0.731
Maintenance agent^b^ Halothane/Isoflorane	19/11	19/11	18/12	0.954
Surgery duration (min)^a^	105.16 ± 18.91	101 ± 19	102.3 ± 17.86	0.598
Anesthesia duration (min)^a^	119.66 ± 17.66	116.5 ± 8.34	113.83 ± 15.46	0.573
Surgical incision length	15 (14–16)	15 (15–17)	14 (15–17)	0.765

a Values that were expressed by Mean ± SD

b Values that were expressed by number/proportion.

The hemodynamic response between groups.

The hemodynamic response including both pulse rate and mean arterial pressure was comparable between groups before incision, after incision, and at 20 min, 40 min, and 60 min PACU (Table 2).

**Table 2:** Comparison of hemodynamic response between the groups.

Hemodynamic Response		Group CA	Group CP	Group CD	P-value
Before incision	PR MAP	121.7 ± 8.38 62.93 ± 3.18	122.56 ± 10.3664.4 ± 3.81	124.83 ± 8.09 64.26 ± 3.31	0.384 0.196
After incision	PR MAP	130.16 ± 7.6 65.93 ± 3.17	166.4 ± 3.89 6.26 ± 8.89	129.5 ± 9.25 65.36 ± 3.20	0.264 0.509
Arrival at PACU	PR MAP	135.23 ± 7.75 66.7 ± 2.66	131.66 ± 8.6367.06 ± 3.18	132.7 ± 9.41 66.03 ± 2.51	0.509 0.350
At 20 min PACU	PR MAP	126.43 ± 7.66 65.26 ± 2.75	124.66 ± 8.6365.6 ± 3.47	123.8 ± 9.73 65.18 ± 3.04	0.494 0.516
At 40 min PACU	PR MAP	121.46 ± 7.1 64.43 ± 2.71	121.5 ± 8.53 64.86 ± 2.94	119.13 ± 9.49 64.03 ± 2.55	0.498 0.503
At 60 min PACU	PR MAP	123.06 ± 9.43 64.1 ± 2.36	119.5 ± 6.98 64.53 ± 2.88	116.06 ± 9.49 63.7 ± 2.45	0.197 0.460

N·B PACU: Post-Anesthesia Care Unit.

### 3.2 Postoperative NRS/FLACC score between groups

**Fig. 1:**
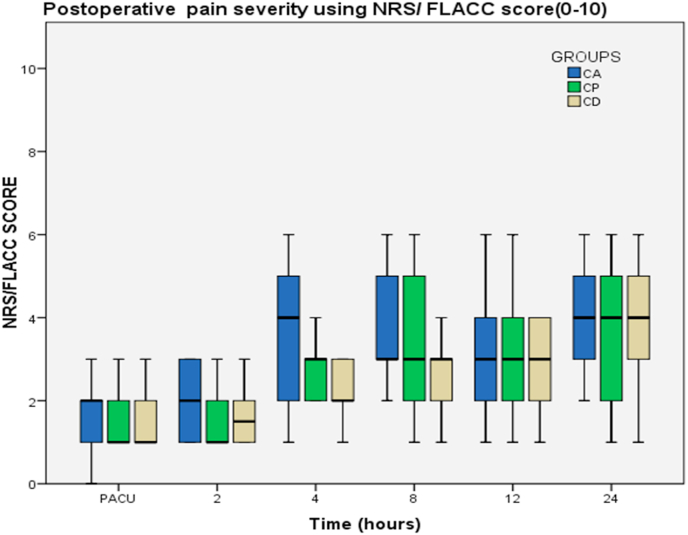
Comparison of postoperative pain score (NRS/FLACC score).

The Kruskal-Wallis test showed that the median of the NRS/FLACC score was not significant at 1st, 2nd, 12th, and 24th hours (*p* > 0.05) between the three groups. There were statistically significant difference results at 4th and 8th hours between groups (p = 0.001 & 0.012) respectively. Post hoc analysis reveals a significant reduction of pain score in CD group compared to CP and CA group at both 4th and 8th hour with p = 0.007, <0.001 & p < 0.032, = 0.003 respectively. Again, there was a statistically significant difference between CP and the CA group at the 4th hour with p = 0.026. However, there was a lower median value in CP than the CA group it's not statistically significant with p > 0.05 (Fig. 1).

### 3.3 Time to first analgesic request and a total of 24 h analgesics consumption

There was a statistically significant difference between the groups with a p-value of <0.001 in time to the first analgesic requirement as expressed in Median (IQR) were 315(210–720), 445(240–840), 735(540–1200) for CA, CP, and CD respectively. Total postoperative analgesia consumption within 24 h between groups. Post hoc analysis of total rectal paracetamol consumption in 24 h showed significantly higher in the caudal alone group when compared to CP and CD with p-values of p < 0.001. While the median rectal paracetamol consumption was higher in CP when compared with a CD with a p-value of 0.013.

### 3.4 Frequency of analgesia request between groups

Every study participant at least requests analgesia once within 24 h postoperatively. From Post hoc analysis there was a significant reduction of the frequency of analgesia request in CD group 1.5 (1–3) compared to CP 3 (1–4), and CA groups 3 (2–4) with p-values of <0.001 respectively. Again, the frequency of analgesic requests in the CP group was lower than the CA group with a p-value of 0.016.

### 3.5 Incidence of nausea and vomiting

**Fig. 2:**
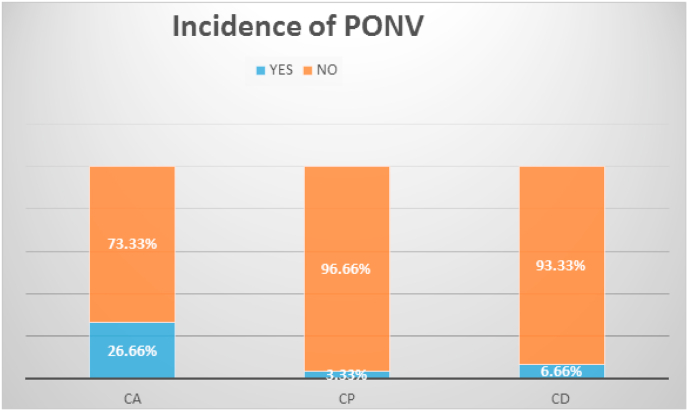
Incidence of Postoperative nausea and vomiting between groups.

There was a statistically significant increase in the incidence of nausea and vomiting over 24 h in CA when compared to the CD and CP groups with p-values of 0.012 and 0.015 respectively. No serious complications or life-threatening events occurred in all groups within 24 h (Fig. 2).

## 4 Discussion

In our study, demographic, and baseline clinical characteristics including hemodynamic variables between groups were comparable. So, the difference in regards to the severity of pain, duration of analgesia, total analgesic consumption along with the frequency of analgesia request, and incidence of adverse effects within 24 h postoperative period was likely due to rectal diclofenac and rectal paracetamol effects in the exposed groups.

This study showed a significant difference in the median pain severity score at the 4th and 8th hours between the groups. Median pain severity score was significantly lower in the CD group compared to CP and CA groups at 4th hours with p-values of = 0.007, <0.001, and at 8th hour with p-values of 0.032, 0.003 respectively. Again, pain scores in the CP group were lower compared to the CA group at the 4th hour with a p-value of 0.026. These results are in line with a study done in Nigeria [10] while it is contrary to a study done by Ozyuvaci.et al. [9]. A possible reason might be the local anesthetic dosage difference used.

With regards to analgesia duration, in our study we observed a lower median time to request the first analgesia in CA group 315 (240–720) compared to CP 445 (240–840) and CD group 735 (540–1200) minutes), with p-values of <0.001. Similarly, studies were done by Nnaji [10], KanchanamalaB [14], and L. Raghavan [13] showed a lower mean time to request the first analgesia in the CA group when compared with CD and CP groups.

With regards to total postoperative analgesic consumption, we observed lower rescue analgesic consumption in the CD group with p-values of <0.001 which is similar to studies done in India [13,15]. different studies in pediatric surgical procedures observed lower postoperative total analgesic consumption in rectal diclofenac compared to rectal paracetamol group with P-values of 0.05 which is consistent with our finding [19,20].

Based on a study done by Hosseini Jahromi SA.et al. [21] a comparative pain score, time to request the first analgesia, and postoperative analgesia consumption between caudal alone and incision or wound site infiltration were observed in pediatric patients undergoing the infra-umbilical procedure. Similarly, multiple studies also found superior analgesia effectiveness of rectal diclofenac or rectal paracetamol combined with wound site infiltration compared to wound site infiltration alone group as it improves analgesia quality in the caudal block [22,23].

This study found the incidence of postoperative nausea vomiting is 3.33% in the CP group, 6.66% in the CD group, and 26.66% in the CA group. However, there is no published study that compares the incidence of PONV between CD and CP group our study demonstrates comparable effect between them. This reduction in incidences of PONV in CD and CP groups might be due to effective analgesia secured from drugs combined with caudal block as pain is expected to increase anxiety and PONV.

## 5 Conclusion

The FLACC/NRS score recorded was significantly reduced in addition to prolonged-time to request the first analgesia in the CD group compared to CA and CP group. Furthermore, the CD group showed lower postoperative analgesic consumption. Based on our findings we recommend the consideration of caudal block combined with rectal diclofenac for infra umbilical surgery in pediatrics.

## Availability of data

Data are available from the first author based on reasonable request.

## Trial registry number

1. Name of the registry: http://www.researchregistry.com
2. Unique Identifying number or registration ID: researchregistry6238
3. Hyperlink to your specific registration (must be publicly accessible and will be checked): https://www.researchregistry.com/browse-the registry#home/registrationdetails/5fa9102a5030b800153ebb76/

## Guarantor

Mr. Diriba Teshome, Mr. Dereje Zewdu.

## Sources of funding

10.13039/501100007941Addis Ababa University.

## Ethical approval

Ethical clearance was obtained from Addis Ababa University ethical clearance committee and Confidentiality of the information were assured by using code numbers than personal identification like names and keeping questionnaires locked in a secured place.

## Authors' contributions

Dereje Zewdu performed the inception, design, analysis, interpretation, and drafting of a research manuscript. Misrak WoldeYohannis, Fissiha Fentie, Abdisa Aga, Assefa Hika, and Diriba Teshome also contributed to the analysis, interpretation, and drafting of the research manuscript. All authors read and approved the revised manuscript for publication.

## Provenance and peer review

Not commissioned, externally peer reviewed.

## Declaration of competing interest

The authors report no conflict of interest.

## Acronyms and abbreviations

ASA: American Society of Anesthesiology
CA: Caudal alone
CD: Caudal with rectal Diclofenac
CP: Caudal with rectal paracetamol
FLACC/NRS: Face, Legs, Activity, Cry, and Consolability/Numeric Rating scale
GA: General Anesthesia
MAP: Mean Arterial blood Pressure
PR: Pulse Rate

## Annals of Medicine and surgery

The following information is required for submission. Please note that failure to respond to these questions/statements will mean your submission will be returned. If you have nothing to declare in any of these categories then this should be stated.

Please state any conflicts of interest.

All authors must disclose any financial and personal relationships with other people or organisations that could inappropriately influence (bias) their work. Examples of potential conflicts of interest include employment, consultancies, stock ownership, honoraria, paid expert testimony, patent applications/registrations, and grants or other funding.

Nothing to declare.

Please state any sources of funding for your research.

All sources of funding should be declared as an acknowledgement at the end of the text. Authors should declare the role of study sponsors, if any, in the collection, analysis and interpretation of data; in the writing of the manuscript; and in the decision to submit the manuscript for publication. If the study sponsors had no such involvement, the authors should so state.

Nothing to declare.

Ethical Approval.

Research studies involving patients require ethical approval. Please state whether approval has been given, name the relevant ethics committee and the state the reference number for their judgement.

Ethical clearance was obtained from Addis Ababa University ethical clearance committee.
